# Comparative Risk of Major Bleeding With Concomitant Use of Oral Anticoagulants and Corticosteroid Bursts

**DOI:** 10.1111/cts.70311

**Published:** 2025-07-26

**Authors:** Tsung‐Chieh Yao, Sheng‐Mao Chang, Yi‐Fen Tsai, Shuo‐Ju Chiang, Hui‐Ju Tsai

**Affiliations:** ^1^ Division of Allergy, Asthma, and Rheumatology, Department of Pediatrics Chang Gung Memorial Hospital Taoyuan Taiwan; ^2^ School of Medicine Chang Gung University College of Medicine Taoyuan Taiwan; ^3^ Department of Statistics National Taipei University Taipei Taiwan; ^4^ Institute of Population Health Sciences National Health Research Institutes Zhunan Taiwan; ^5^ Division of Cardiology, Department of Internal Medicine Taipei City Hospital Taipei Taiwan; ^6^ Graduate Institute of Biomedical Materials and Tissue Engineering Taipei Medical University Taipei Taiwan; ^7^ Department of Medical Science National Tsing‐Hua University Hsinchu Taiwan

**Keywords:** atrial fibrillation, major bleeding, non–vitamin K anticoagulant, oral corticosteroid, warfarin

## Abstract

The choice of oral anticoagulants and oral corticosteroid (OCS) burst cotherapy may influence the risk of major bleeding; however, this risk remains poorly characterized. We aimed to quantify the comparative safety of non–vitamin K oral anticoagulants (NOACs) versus warfarin on major bleeding while receiving OCS burst cotherapy among patients with atrial fibrillation. A nationwide population‐based cohort study was conducted using the National Health Insurance Research Database. We examined associations between NOACs (edoxaban, apixaban, dabigartran, or rivaroxaban) or warfarin with OCS burst cotherapy and major bleeding. We measured the risk by estimating incidence, incidence risk ratios (IRRs), and adjusted hazard ratios (AHRs) after adjusting for baseline differences using overlap weighting. In this study, among 239,693 patients receiving oral anticoagulants, 50,390 (21%) received at least one OCS burst, defined as OCS use for less than 30 days, were included. A lower risk of major bleeding related to OCS burst cotherapy with NOACs versus warfarin was noted (AHR = 0.57 [95% CI = 0.52–0.61]). The greatest incidence was observed in patients with warfarin and OCS burst cotherapy (67.30 per 1000 person‐years). The incidence for patients prescribing OCS burst cotherapy with edoxaban (30.36 per 1000 person‐years; IRR = 0.45 [95% CI = 0.38–0.53]), apixaban (34.93 per 1000 person‐years; IRR = 0.52 [95% CI = 0.45–0.60]), dabigatran (42.47 per 1000 person‐years; IRR = 0.63 [95% CI = 0.56–0.72]), and rivaroxaban (46.99 per 1000 person‐years; IRR = 0.70 [95% CI = 0.63–0.77]), separately, was lower than that with warfarin. The results reveal that the incidence of major bleeding was lowest for edoxaban and highest for warfarin, with notable differences in incidence rates across NOACs among patients receiving oral anticoagulants and OCS burst cotherapy.

**Summary**

- What is the current knowledge on the topic?
  ○ The choice of oral anticoagulants and oral corticosteroid (OCS) burst cotherapy may influence the risk of major bleeding.
- What question did this study address?
  ○ What is the comparative risk of major bleeding associated with different oral anticoagulants among patients with atrial fibrillation receiving OCS burst cotherapy?
- What does this study add to our knowledge?
  ○ In this national population‐based cohort study of 239,693 patients with atrial fibrillation on oral anticoagulation, 50,390 (21%) received at least one OCS burst cotherapy. Among these patients, the incidence rate of major bleeding was lowest for edoxaban, followed by apixaban, dabigatran, and rivaroxaban, and highest for warfarin.
- How might this change clinical pharmacology or translational science?
  ○ These findings highlight the importance of prudent use of oral anticoagulants and OCS burst cotherapy and might guide the selection of oral anticoagulants for patients with atrial fibrillation.

## 1 Introduction

Atrial fibrillation is known to increase the risk of stroke, resulting in nearly 15%–20% of all strokes [1, 2, 3, 4]. Anticoagulation is crucial to prevent the occurrence of ischemic strokes among patients with atrial fibrillation [5]. Non–vitamin K oral anticoagulants (NOACs) have a superior safety profile compared to warfarin because of their predictable anticoagulant effect, convenient dosing, and comparative efficacy in diminishing thromboembolism and major bleeding [1, 6]. However, NOACs may still cause a risk of major bleeding among patients with atrial fibrillation, particularly in the presence of polypharmacy or multimorbidity [7, 8].

Polypharmacy is prevalent in patients with atrial fibrillation and may impact treatment effectiveness and cause adverse events [9]. Observational studies have assessed the association of cotherapy of oral anticoagulants and other medications with the risk of bleeding events [10, 11]. For example, a retrospective cohort study in the US has suggested a lower risk of gastrointestinal bleeding among patients receiving oral anticoagulants with proton pump inhibitors (PPIs) cotherapy [10]. A nested case–control study has reported that cotherapy with selective serotonin reuptake inhibitors (SSRIs) was associated with an elevated risk of major bleeding [11]. Recent nationwide studies in the US and Taiwan have documented that oral corticosteroid (OCS) burst increased risks of gastrointestinal bleeding, heart failure, sepsis, and venous thromboembolism, etc. [12, 13, 14]. Previous studies have also suggested positive associations between patients using high‐dose OCS therapy and an increased risk of developing atrial fibrillation [15, 16]. To our knowledge, large real‐world observational studies investigating the adverse clinical outcomes of oral anticoagulants and OCS burst (defined as OCS continuous use for 30 days or less after initiation) cotherapy among patients with atrial fibrillation, a vulnerable population, are lacking.

This study addresses the knowledge gaps using a nationwide cohort study to quantify the comparative safety of OCS burst cotherapy with various oral anticoagulants on major bleeding among patients with atrial fibrillation.

## 2 Methods

### 2.1 Data Source

We used the data derived from the entire Taiwan's National Health Insurance Research Database (NHIRD) from January 2010 through December 2021. NHIRD comprises the medical and pharmacy claims data of approximately 23.6 million participants, representing roughly 99% of Taiwan's population. Detailed information of NHIRD has been described elsewhere [17, 18]. The study was approved by the Institutional Review Board of the National Health Research Institutes, Taiwan (EC1090702‐E). Written informed consent was waived because all data were encrypted.

### 2.2 Study Designs and Populations

**FIGURE 1:**
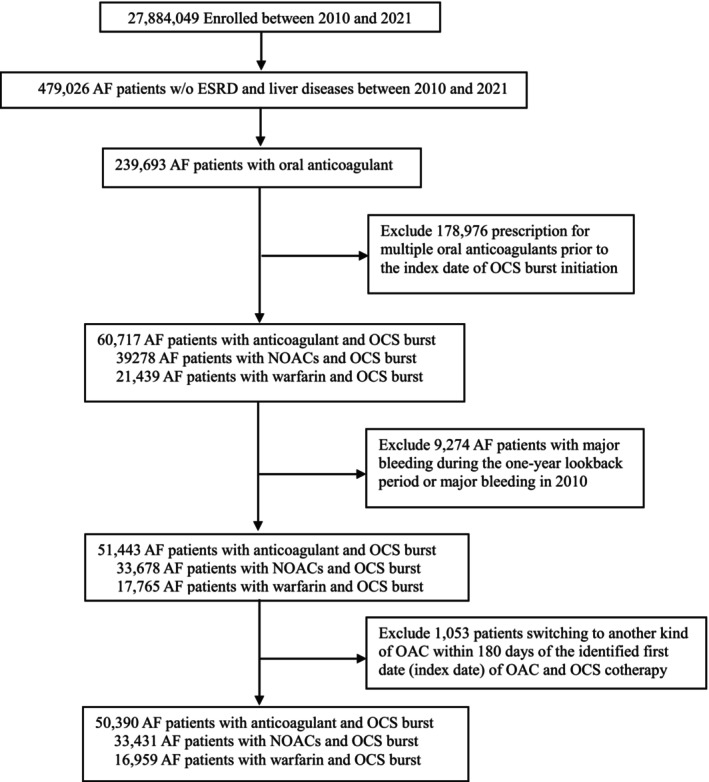
Flow chart of patients identification. AF, atrial fibrillation; ESRD, end‐stage renal disease; NOACs, non–vitamin K anticoagulants; OAC, oral anticoagulant; OCS, oral corticosteroid.

We employed a nationwide cohort to estimate the comparative safety of oral anticoagulants and OCS burst cotherapy with major bleeding. The study population was patients with atrial fibrillation, defined as those with 1 inpatient or 2 consecutive outpatient records of atrial fibrillation diagnosis (the *International Classification of Diseases, Ninth Revision, Clinical Modification* (*ICD‐9‐CM*) *codes* 427.31, 427.32 and the *International Classification of Diseases, Tenth Revision, Clinical Modification* (*ICD‐10‐CM*) *code* I48) [19]. Patients with end‐stage renal disease and liver diseases were excluded for subsequent analyses. The flowchart of patients' identification was depicted in Figure 1.

In this study, we categorized patients with atrial fibrillation into two groups based on their anticoagulant medication type: NOACs and warfarin groups. The NOACs group was defined as patients prescribing four specific NOACs, that is, edoxaban, apixaban, dabigatran, or rivaroxaban; and the warfarin group was defined as those treated with warfarin. Patients treated with oral anticoagulants and OCS burst cotherapy were included. Patients with major bleeding during the one‐year lookback period or prescription for multiple oral anticoagulants were excluded (Figure 1).

### 2.3 Exposure and Outcome Assessment

The exposure was oral anticoagulants and OCS burst cotherapy. We first identified patients who were prescribed oral anticoagulants. We defined exposure as patients prescribed an OCS burst, defined as OCS continuous use for 30 days or less after initiation, during their treatment with oral anticoagulants. Cohort entry date was defined as the date of the indications prescription with the presence of oral anticoagulants. The dosages of OCS were standardized to prednisone equivalent dose and examined in this study accordingly (Table S1).

The primary and secondary outcomes of interest were major bleeding and gastrointestinal bleeding, defined using *ICD‐9‐CM codes* prior to 2015 and *ICD‐10‐CM codes* thereafter (Table S2). Follow‐up began on the day of OCS burst prescription and continued for 365 days or until 31 December 2021.

### 2.4 Covariates

The covariates were considered as follows: (1) demographic characteristics (e.g., age, sex, residence, and income level); (2) comorbid conditions (e.g., Charlson's comorbidity index score, myocardial infarction, congestive heart failure, peripheral vascular disease, stroke, transient ischemic attack, dementia, chronic pulmonary disease, anemia, kidney diseases, hepatic diseases, hypertension, bleeding history, and alcohol use); (3) concomitant medication use (e.g., non‐steroidal anti‐inflammatory drugs (NSAIDs), proton pump inhibitors, antihypertensives, clopidogrel, ticlopidine, insulin, oral hypoglycemic agents, and lipid lowering agents); and (4) healthcare utilization (e.g., total number of inpatient and outpatient visits). Data from the 12 months prior to the first eligible date of cotherapy were adjusted in the subsequent analyses.

### 2.5 Statistical Analysis

We employed overlap weighting, a propensity‐score‐based approach, to balance baseline characteristics and minimize potential confounding effects [20]. Particularly, we computed the propensity score using a logistic regression model which included all variables provided in the above “Covariates” subsection, followed by estimating overlap weights of each patient. This approach upweighted patients in the overlapping portion of the propensity‐score distribution through giving each exposed patient a weight reflecting the probability of being assigned to the other group [21]. We plotted the propensity‐score distributions before and after overlap weighting. The plots suggested the representativeness of the study population (Figure S2). We computed standardized mean difference (SMD) to assess the differences in baseline characteristics between two groups [22]. We calculated the adjusted incidence rates per 1000 person‐years of major bleeding in patients with oral anticoagulants (e.g., edoxaban, apixaban, dabigatran, rivaroxaban and warfarin) and OCS burst cotherapy. Risk difference (RD) and incidence risk ratios (IRRs) were calculated accordingly. We applied Cox proportional hazards regression models to evaluate the comparative risk of major bleeding on OCS burst cotherapy with NOACs versus warfarin using a 365‐day follow‐up period. Adjusted hazard ratios (AHRs) with 95% confidence interval (CI) were used to quantify the comparative risk of major bleeding. Follow‐up terminated on whichever of the following events came first: occurrence of major bleeding; loss of follow‐up; the end of the follow‐up period; or death. Kaplan–Meier plots were created to estimate the cumulative incidence of major bleeding between patients receiving OCS burst cotherapy with NOACs versus warfarin.

Sensitivity analyses were performed to evaluate the potential influence of different follow‐up periods: 90 and 180 days; exclusion of patients aged more than 80 years; exclusion of patients with kidney diseases; and exclusion of patients with intravenous or long‐term inhaled corticosteroid treatments. To examine whether Cox proportional hazards assumptions were valid, we added product terms between predictors and survival function in the models, and plotted Kaplan–Meier curves with 1‐year follow‐up time versus survival function. No significant product terms were found, and the parallel Kaplan–Meier curves were observed (Figure S1), suggesting assumptions were held. *p* < 0.05 was considered statistically significant. All analyses were carried out using SAS version 9.4 (SAS Institute, Cary, NC, USA). AI tools were not used for drafting the manuscript or developing figures and tables.

## 3 Results

### 3.1 Baseline Characteristics

Between January 1, 2010, and December 31, 2021, a total of 239,693 patients with atrial fibrillation treated with oral anticoagulants were identified, of whom 21% (*n* = 50,390) received at least 1 OCS burst during a mean follow‐up of 0.92 (SD, 0.23) years. Among those, 33,431 patients were treated with NOACs and 16,959 were treated with warfarin during the 12‐year study period. Table 1 shows the baseline and clinical characteristics of study patients. After overlap weighting, the SMDs indicated good balance regarding baseline and clinical characteristics between patients receiving NOACs and OCS burst cotherapy and those receiving warfarin and OCS burst cotherapy (Table 1). The top 10 indications for OCS burst, including the number of patients and the proportion of each indication, were shown in Table S3.

**TABLE 1:** Baseline characteristics in patients with atrial fibrillation in the cohort study.

	All	NOACs + OCS burst	Warfarin + OCS burst	SMD^1^	SMD^2^
	*n* = 50,390	*n* = 33,431	*n* = 16,959		
Age, mean (SD)	73.84 (11.11)	75.62 (10.12)	70.34 (12.10)	0.47	0.00
Sex, *n* (%)					
Male	28,520 (56.60)	19,118 (57.19)	9402 (55.44)	0.04	0.00
Female	21,870 (43.40)	14,313 (42.81)	7557 (44.56)	0.04	0.00
Residence, *n* (%)					
Urban group low	8069 (16.05)	5422 (16.26)	2647 (15.64)	0.02	0.00
Urban group medium	16,866 (33.55)	11,015 (33.03)	5851 (34.56)	0.03	0.00
Urban group high	25,342 (50.40)	16,912 (50.71)	8430 (49.80)	0.02	0.00
Insurance amount, TWD, *n* (%)					
0–20,000	15,320 (30.40)	9741 (29.14)	5579 (32.90)	0.08	0.00
20,000–40,000	26,376 (52.34)	17,795 (53.23)	8581 (50.60)	0.05	0.00
≥ 40000	8694 (17.25)	5895 (17.63)	2799 (16.50)	0.03	0.00
CCI score, mean (std)	2.67 (2.14)	2.78 (2.17)	2.47 (2.06)	0.15	0.00
CCI score, median (q1‐q3)	2 (1–4)	2 (1–4)	2 (1–4)		
Myocardial infarction, *n* (%)					
No	48,350 (95.95)	32,048 (95.86)	16,302 (96.13)	0.01	0.00
Yes	2040 (4.05)	1383 (4.14)	657 (3.87)	0.01	0.00
Congestive heart failure, *n* (%)					
No	30,004 (59.54)	20,188 (60.39)	9816 (57.88)	0.05	0.00
Yes	20,386 (40.46)	13,243 (39.61)	7143 (42.12)	0.05	0.00
Peripheral vascular disease, *n* (%)					
No	47,679 (94.62)	31,714 (94.86)	15,965 (94.14)	0.03	0.00
Yes	2711 (5.38)	1717 (5.14)	994 (5.86)	0.03	0.00
Stroke, *n* (%)					
No	39,763 (78.91)	26,202 (78.38)	13,561 (79.96)	0.04	0.00
Yes	10,627 (21.09)	7229 (21.62)	3398 (20.04)	0.04	0.00
Transient ischemic attack, *n* (%)					
No	48,004 (95.26)	31,756 (94.99)	16,248 (95.81)	0.04	0.00
Yes	2386 (4.74)	1675 (5.01)	711 (4.19)	0.04	0.00
Dementia, *n* (%)					
No	46,368 (92.02)	30,398 (90.93)	15,970 (94.17)	0.12	0.00
Yes	4022 (7.98)	3033 (9.07)	989 (5.83)	0.12	0.00
Chronic pulmonary disease, *n* (%)					
No	31,590 (62.69)	20,841 (62.34)	10,749 (63.38)	0.02	0.00
Yes	18,800 (37.31)	12,590 (37.66)	6210 (36.62)	0.02	0.00
Anemia, *n* (%)					
No	46,794 (92.86)	30,990 (92.70)	15,804 (93.19)	0.02	0.00
Yes	3596 (7.14)	2441 (7.30)	1155 (6.81)	0.02	0.00
Kidney diseases, *n* (%)					
No	40,202 (79.78)	25,971 (77.69)	14,231 (83.91)	0.16	0.00
Yes	10,188 (20.22)	7460 (22.31)	2728 (16.09)	0.16	0.00
Hepatic diseases, *n* (%)					
No	47,058 (93.39)	31,341 (93.75)	15,717 (92.68)	0.04	0.00
Yes	3332 (6.61)	2090 (6.25)	1242 (7.32)	0.04	0.00
Hypertension, *n* (%)					
No	15,582 (30.92)	9137 (27.33)	6445 (38.00)	0.23	0.00
Yes	34,808 (69.08)	24,294 (72.67)	10,514 (62.00)	0.23	0.00
Alcohol use, *n* (%)					
No	50,268 (99.76)	33,344 (99.74)	16,924 (99.79)	0.01	0.00
Yes	122 (0.24)	87 (0.26)	35 (0.21)	0.01	0.00
Number of outpatient visits, mean (SD)	42.87 (23.45)	43.05 (23.68)	42.51 (22.97)	0.02	0.00
Number of outpatient visits, median (IQR)	38 (26–55)	38 (26–55)	38 (26–54)		
Number of inpatient visits, mean (SD)	0.98 (1.56)	1.01 (1.59)	0.91 (1.50)	0.06	0.00
Number of inpatient visits, median (IQR)	0 (0–1)	0 (0–1)	0 (0–1)		
NSAIDs, *n* (%)					
No	17,797 (35.32)	14,228 (37.54)	5085 (29.98)	0.17	0.00
Yes	32,593 (64.68)	23,671 (62.46)	11,874 (70.02)	0.17	0.00
Proton pump inhibitors, *n* (%)					
No	42,513 (84.37)	30,537 (80.57)	15,034 (88.65)	0.18	0.00
Yes	7877 (15.63)	7362 (19.43)	1925 (11.35)	0.18	0.00
Antihypertensives, *n* (%)					
No	16,256 (32.26)	14,768 (38.97)	3106 (18.31)	0.48	0.00
Yes	34,134 (67.74)	23,131 (61.03)	13,853 (81.69)	0.48	0.00
Clopidogrel, *n* (%)					
No	47,028 (93.33)	34,522 (91.09)	16,523 (97.43)	0.27	0.00
Yes	3362 (6.67)	3377 (8.91)	436 (2.57)	0.27	0.00
Ticlopidine, *n* (%)					
No	49,880 (98.99)	37,416 (98.73)	16,837 (99.28)	0.05	0.00
Yes	510 (1.01)	483 (1.27)	122 (0.72)	0.05	0.00
Insulin, *n* (%)					
No	47,136 (93.54)	35,116 (92.66)	16,178 (95.39)	0.12	0.00
Yes	3254 (6.46)	2783 (7.34)	781 (4.61)	0.12	0.00
Oral hypoglycemic agents, *n* (%)					
No	39,736 (78.86)	28,363 (74.84)	14,767 (87.07)	0.32	0.00
Yes	10,654 (21.14)	9536 (25.16)	2192 (12.93)	0.32	0.00
Lipid lowering agents, *n* (%)					
No	34,453 (68.37)	22,793 (60.14)	14,375 (84.76)	0.57	0.00
Yes	15,937 (31.63)	15,106 (39.86)	2584 (15.24)	0.57	0.00

*Note:* SMD^1^ is computed before overlap weighting. SMD^2^ is computed after overlap weighting.

Abbreviations: CCI, Charlson Comorbidity Index; IQR, interquartile range; NOAC, non–vitamin K anticoagulant; NSAIDs, non‐steroidal anti‐inflammatory drugs; OCS, oral corticosteroid; SD, standard deviation; SMD, standardized mean difference; TWD, Taiwanese new dollar.

### 3.2 Comparative Risk of Major Bleeding

**FIGURE 2:**
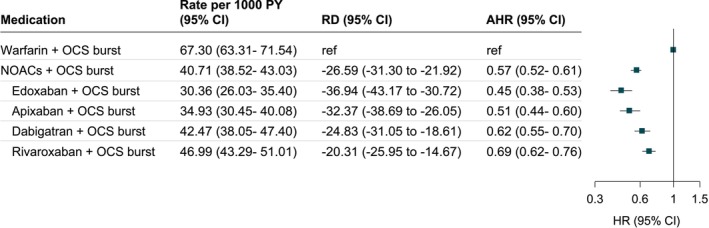
Association of oral anticoagulants and OCS burst cotherapy with major bleeding in patients with atrial fibrillation during 365‐day follow‐up period. AHR, adjusted hazard ratio; CI, confidence interval; NOACs, non–vitamin K anticoagulants; OCS, oral corticosteroid; RD, risk difference. Model was adjusted for age, sex, residence, income level, Charlson comorbidity index score, myocardial infarction, congestive heart failure, peripheral vascular disease, stroke, transient ischemic attack, dementia, chronic pulmonary disease, anemia, kidney diseases, and hepatic diseases, hypertension, bleeding history, and alcohol use, number of outpatient visits, non‐steroidal anti‐inflammatory drugs, proton pump inhibitors, antihypertensives, clopidogrel, ticlopidine, insulin, oral hypoglycemic agents, and lipid lowering agents.

**FIGURE 3:**
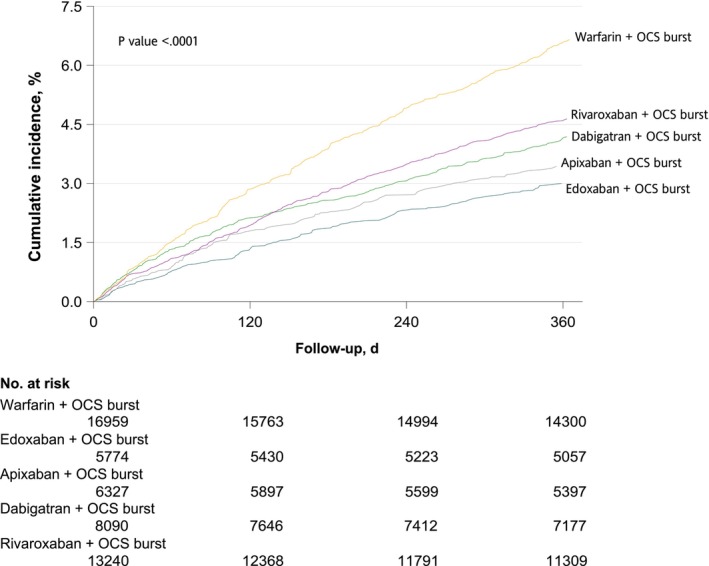
Oral anticoagulants and OCS burst cotherapy and cumulative incidence of major bleeding during the 365‐day follow‐up period. OCS, oral corticosteroid.

We evaluated the comparative risks of major bleeding and gastrointestinal bleeding, separately, with concomitant use of NOACs versus warfarin and OCS burst cotherapy. The results suggested that AHRs of major bleeding and gastrointestinal bleeding during the 365‐day follow‐up period were all significantly lower in patients receiving OCS burst cotherapy with NOACs than those receiving warfarin (AHR: 0.57; 95% CI: 0.52–0.61 for major bleeding; and AHR: 0.64; 95% CI: 0.58–0.70 for gastrointestinal bleeding; Figure 2 and Figure S3). When further scrutinizing each individual NOAC versus warfarin, Figures 2 and 3 reveal significantly lower risks of major bleeding were found in patients receiving OCS burst cotherapy with edoxaban, apixaban, rivaroxaban, and dabigatran than those receiving warfarin (AHR, 0.45 [95% CI, 0.38–0.53] for edoxaban; AHR, 0.51 [95% CI, 0.44–0.60] for apixaban; AHR, 0.62 [95% CI, 0.55–0.70] for dabigatran; and AHR, 0.69 [95% CI, 0.62–0.76] for rivaroxaban). We also evaluated the dose–response relationship using OCS median daily dose of 10 mg as a cutoff. No dose–response relationship was observed (AHR: 0.57; 95% CI: 0.52–0.64 for daily dose ≤ 10 mg; AHR: 0.55; 95% CI: 0.49–0.63 for daily dose > 10 mg; Table S4).

### 3.3 Individual Anticoagulants and OCS Burst Cotherapy

For patients receiving various oral anticoagulants and OCS burst cotherapy, the overall adjusted incidence of major bleeding was 49.54 [95% CI, 47.55 to 51.61] per 1000 person‐years. Table 2 presents the adjusted incidences of major bleeding for OCS burst cotherapy with edoxaban, apixaban, dabigatran, rivaroxaban, and warfarin, respectively. The greatest incidence was observed in patients with warfarin and OCS burst cotherapy (67.30 per 1000 person‐years). The incidence for patients with edoxaban and OCS burst cotherapy (30.36 per 1000 person‐years) was significantly lower than the incidence for those receiving OCS burst cotherapy with dabigatran (IRR, 0.71 [95% CI, 0.59–0.86]; RD, −12.11 [95% CI, −18.71 to −5.51]), rivaroxaban (IRR, 0.65 [95% CI, 0.54–0.77]; RD, −16.64 [95% CI, −22.69 to −10.58]), and warfarin (IRR, 0.45 [95% CI, 0.38–0.53]; RD, −36.94 [95% CI, −43.17 to −30.72]), but not for apixaban. The incidence for patients with apixaban and OCS burst cotherapy (34.93 per 1000 person‐years) was significantly lower than those taking OCS burst cotherapy with dabigatran (IRR, 0.82 [95% CI, 0.69–0.98]; RD, −7.54 [95% CI, −14.23 to −0.84]), rivaroxaban (IRR, 0.74 [95% CI, 0.63–0.87]; RD, −12.06 [95% CI, −18.22 to −5.90]), and warfarin (IRR, 0.52 [95% CI, 0.45–0.60]; RD, −32.37 [95% CI, −38.69 to −26.05]). The incidence for patients with dabigatran and OCS burst cotherapy (42.47 per 1000 person‐years) was significantly lower than the incidence for those prescribing OCS burst cotherapy with warfarin (IRR, 0.63 [95% CI, 0.56–0.72]; RD, −24.83 [95% CI, −31.05 to −18.61]). The incidence for patients with rivaroxaban and OCS burst cotherapy (46.99 per 1000 person‐years) was significantly lower than the incidence for OCS burst cotherapy with warfarin (IRR, 0.70 [95% CI, 0.63–0.77]; RD, −20.31 [95% CI, −25.95 to −14.67]).

**TABLE 2:** Comparative incidence of major bleeding for patients receiving various oral anticoagulants and OCS burst cotherapy in the cohort study.

	Major bleeding	Person‐years	Adjusted incidence/1000 person‐years (95% CI)
Edoxaban + OCS burst	166	5342	30.36 (26.03–35.40)
Apixaban + OCS burst	202	5771	34.93 (30.45–40.08)
Dabigatran + OCS burst	319	7550	42.47 (38.05–47.40)
Rivaroxaban + OCS burst	566	12,104	46.99 (43.29–51.01)
Warfarin + OCS burst	1198	15,417	67.30 (63.31–71.54)
		**RD (95% CI)**	**IRR (95% CI)** ^a^
Edoxaban + OCS burst vs.			
Apixaban + OCS burst		−4.58 (−11.27 to 2.12)	0.87 (0.71 to 1.07)
Dabigatran + OCS burst		−12.11 (−18.71 to −5.51)	**0.71 (0.59 to 0.86)**
Rivaroxaban + OCS burst		−16.64 (−22.69 to −10.58)	**0.65 (0.54 to 0.77)**
Warfarin + OCS burst		−36.94 (−43.17 to −30.72)	**0.45 (0.38 to 0.53)**
Apixaban + OCS burst vs.			
Dabigatran + OCS burst		−7.54 (−14.23 to −0.84)	**0.82 (0.69 to 0.98)**
Rivaroxaban + OCS burst		−12.06 (−18.22 to −5.90)	**0.74 (0.63 to 0.87)**
Warfarin + OCS burst		−32.37 (−38.69 to −26.05)	**0.52 (0.45 to 0.60)**
Dabigatran + OCS burst vs.			
Rivaroxaban + OCS burst		−4.52 (−10.58 to 1.53)	0.90 (0.79 to 1.04)
Warfarin + OCS burst		−24.83 (−31.05 to −18.61)	**0.63 (0.56 to 0.72)**
Rivaroxaban + OCS burst vs.			
Warfarin + OCS burst		−20.31 (−25.95 to −14.67)	**0.70 (0.63 to 0.77)**

*Note:* The bold values indicate statistical significance (*p*‐value less than 0.05).

Abbreviations: CI, confidence interval; IRR, incidence risk ratio; OCS, oral corticosteroid; RD, risk difference.

^a^ Model was adjusted by overlap weighting using the propensity score for age, sex, residence, income level, Charlson comorbidity index score, myocardial infarction, congestive heart failure, peripheral vascular disease, stroke, transient ischemic attack, dementia, chronic pulmonary disease, anemia, kidney diseases, hepatic diseases, hypertension, bleeding history, alcohol use, number of outpatient visits, non‐steroidal anti‐inflammatory drugs, proton pump inhibitors, antihypertensives, clopidogrel, ticlopidine, insulin, oral hypoglycemic agents, and lipid lowering agents.

### 3.4 Sensitivity Analyses

Sensitivity analyses were performed for validating and assessing the robustness of main findings; specifically, we assessed the impacts of different follow‐up periods (90 and 180 days) instead of the 1‐year follow‐up period used in the main analyses, excluded patients aged more than 80 years, excluded patients with a history of kidney diseases, and excluded patients with intravenous or long‐term inhaled corticosteroid treatments, respectively. We found AHRs of major bleeding, ranging from 0.55 to 0.64, were all significantly lower in patients receiving OCS burst cotherapy with NOACs than those receiving warfarin, which were comparable to those observed in the main results (Figure S4).

## 4 Discussion

This entire national population‐based study of 239,693 patients with atrial fibrillation taking oral anticoagulants in Taiwan has several main findings. First, OCS burst is commonly prescribed to patients receiving oral anticoagulants for atrial fibrillation in real‐world clinical practice, as 21% of the patients received at least one OCS burst cotherapy during a mean follow‐up of 0.92 years. Secondly, the IRR of major bleeding tended to be lower among patients using NOACs than warfarin (IRR 0.57, 0.52 to 0.61). Third, this study found differential risks of major bleeding related to OCS burst cotherapy across various oral anticoagulants. Specifically, we observed significantly lower risks of major bleeding with edoxaban versus dabigatran, rivaroxaban, or warfarin; with apixaban versus rivaroxaban or warfarin; with dabigatran versus warfarin; and with rivaroxaban versus warfarin among patients co‐prescribing OCS burst. The findings of this study provided novel real‐world evidence showing comparative safety and the associations between OCS burst and major bleeding among patients with atrial fibrillation across various oral anticoagulants in a whole nation population over a 12‐year study period.

The risk of major bleeding associated with the concomitant use of oral anticoagulants and other medications, such as PPIs, SSRIs, diltiazem, amiodarone, fluconazole, rifampin, and phenytoin has been evaluated and reported in previous observational studies [10, 11, 19, 23, 24], but evidence about the risk associated with concomitant use of oral anticoagulants and OCS burst is lacking. Given the recently raised concern about the risk of OCS burst on gastrointestinal bleeding events in the general population [12, 13, 14], a knowledge gap has emerged regarding the potential risk of major bleeding following OCS burst in high‐risk patients under oral anticoagulation. This is the first, to our knowledge, nationwide population‐based study demonstrating the risk of major bleeding associated with co‐prescribing OCS burst and oral anticoagulants, particularly the risk was more pronounced for warfarin. We further revealed differential effects of individual NOACs (e.g., edoxaban, apixaban, dabigatran and rivaroxaban) versus warfarin on major bleeding risk. Our findings were evident that differential risks of major bleeding related to OCS burst cotherapy across individual NOACs might be partly explained by their differences in pharmacokinetic or pharmacodynamic profiles.

Our findings have important clinical implications. First, this study provides new real‐world evidence that OCS bursts are frequently prescribed to one‐fifth of patients with atrial fibrillation receiving oral anticoagulants. Although major bleeding events are relatively rare, the absolute number of affected patients could be significant due to the widespread and increasing use of oral anticoagulants globally. Notably, our findings indicate that OCS bursts are commonly prescribed for self‐limited indications (e.g., skin disorders and upper respiratory tract infections), for which effective non‐steroidal treatments are available. This aligns with our prior study in the general adult population, which found that 25% of healthy adults received OCS bursts during a three‐year period, primarily for the aforementioned indications [13]. When assessing the OCS dose–response relationship on the comparative safety of cotherapy with different oral anticoagulants, no OCS dose–response relationship was noted. Therefore, clinical attention should focus on the comparative safety of different oral anticoagulants when co‐prescribed with OCS bursts. The differential effects of individual oral anticoagulants on major bleeding risk during OCS burst cotherapy, as observed in this study, could serve as a guide for optimizing treatment decisions in clinical practice.

### 4.1 Limitations

This study has several limitations. First, the influence of unmeasured residual confounding effects may still remain. After overlap weighting, patients prescribing OCS burst cotherapy with NOACs and those with warfarin were well balanced at baseline. Thus, the impact of unmeasured confounding effects should be limited. On the other hand, the estimates from overlap weighting apply to the subgroup with substantial probability of receiving either treatment, by down‐weighting the estimates in the tails of the distributions of propensity scores. Thus, the results should be interpreted with caution [20]. Secondly, as in all epidemiologic studies, misclassification bias or coding error is a possibility. We only considered primary diagnoses from inpatients and outpatients medical records. As most resulting misclassification is nondifferential, the influence should underestimate the actual risk and be toward the null. Third, as reported in previous studies, a certain proportion of patients interrupted treatment [25, 26]. We performed the sensitivity analysis by adjusting censoring effects through various follow‐up periods and found largely consistent results with those in the main results. Fourth, previous ENGAGE AF‐TIMI 48 (Effective Anticoagulation With Factor Xa Next Generation in Atrial Fibrillation‐Thrombolysis in Myocardial Infarction Study 48) studies have demonstrated population differences in edoxaban response between Asian and non‐Asian populations [27, 28]. The present study used whole population data from the NHIRD in Taiwan. As such, the findings may or may not be generalizable to other populations. Further validation is needed to replicate the findings across different populations.

## 5 Conclusions

In this entire national population‐based study, among patients with atrial fibrillation receiving oral anticoagulants and OCS burst cotherapy, the incidence of major bleeding was lowest for edoxaban and highest for warfarin, with noted differences in incidence rates across NOACs. These findings suggest the need for prudent use of OCS burst and inform the selection of oral anticoagulants when prescribing to patients with atrial fibrillation.

## Author Contributions

T.‐C.Y. and H.‐J.T. wrote the manuscript; T.‐C.Y. and H.‐J.T. designed the research; T.‐C.Y., S.‐M.C., S.‐J.C., and H.‐J.T. performed the research; S.‐M.C., Y.‐F.T., and H.‐J.T. analyzed the data.

## Disclosure

Disclaimer: This study is based in part on data from the National Health Insurance Research Database provided by the Bureau of National Health Insurance of the Ministry of Health and Welfare, Taiwan. The interpretation and conclusions contained in this article do not represent those of the Bureau of National Health Insurance or the Ministry of Health and Welfare.

## Conflicts of Interest

The authors declare no conflicts of interest.

## Supporting information


**Data S1:** cts70311‐sup‐0001‐DataS1.docx.

## Data Availability

De‐identified participant data used from Taiwan are managed by the Taiwan Ministry of Health and Welfare. No additional data are available.
