# Supplementary material for: Comparative Risk of Major Bleeding With Concomitant Use of Oral Anticoagulants and Corticosteroid Bursts
Source: Clin Transl Sci. 2025 Jul 26;18(8):e70311. doi: 10.1111/cts.70311 (PMC12410366; doi:10.1111/cts.70311)
Supplement: Supplementary file 1 — Data S1: cts70311‐sup‐0001‐DataS1.docx. [file CTS-18-e70311-s001.docx]

**Supplemental Information**

**Comparative Risk of Major Bleeding With Concomitant Use of Oral Anticoagulants and Corticosteroid Bursts**

Tsung-Chieh Yao, MD, PhD, Sheng-Mao Chang, PhD, Yi-Fen Tsai, MS, Shuo-Ju Chiang, MD, PhD, Hui-Ju Tsai, MPH, PhD

**Supplementary Table**

**Table S1.** Equivalent doses of oral corticosteroids examined in this study.

**Table S2.** ICD-9-CM and ICD-10-CM codes of atrial fibrillation and major bleeding.

**Table S3.** Diagnosis of top 10 indications for prescribing OCS burst.

**Table S4.** Association of oral anticoagulants with major bleeding in patients with atrial fibrillation with cotherapy of OCS daily dose <10 mg versus OCS daily does >10 mg.

**Supplementary Figure**

**Figure S1.** Kaplan-Meier curve for checking Cox proportional hazards assumption during 365-day follow-up in patients with atrial fibrillation treated various anticoagulants and OCS burst cotherapy.

**Figure S2.** The distributions of propensity scores computed using baseline covariates before and after overlap weighting between patients with OCS burst cotherapy with NOACs versus warfarin.

**Figure S3.** Association of oral anticoagulant and OCS burst cotherapy with major bleeding and gastrointestinal bleeding in patients with atrial fibrillation.

**Figure S4.** Association of oral anticoagulant and OCS burst cotherapy with major bleeding in patients with atrial fibrillation based on different follow-up periods: 90 and 180 days; patients aged less than 80 years; patients without kidney diseases; and patients without intravenous or long-term inhaled corticosteroid treatments.

**Table S1.** Equivalent doses of oral corticosteroids examined in this study.

| **Corticosteroids** | **Equivalent Dose** | |
| --- | --- | --- |
| Betamethasone | 0.6 | mg |
| Dexamethasone | 0.75 | mg |
| Methylprednisolone | 4 | mg |
| Triamcinolone | 4 | mg |
| Prednisone | 5 | mg |
| Prednisolone | 5 | mg |
| Hydrocortisone | 20 | mg |
| Cortisone | 25 | mg |

**Table S2.** ICD-9-CM and ICD-10-CM codes of atrial fibrillation and major bleeding.

| **Diagnosis** | **ICD-9-CM Code** | **ICD-10-CM Code** |
| --- | --- | --- |
| **Atrial fibrillation** | 427.31, 427.32 | I48.0, I48.1, I48.2, I48.3, I48.4, I48.91, I48.92 |
| **Major bleeding** | 530.7, 531, 531.2, 531.4, 531.6, 532, 532.2, 532.4, 532.6, 533, 533.2, 533.4, 533.6, 534, 534.2, 534.4, 534.6, 569.3, 535.01, 535.11, 535.21, 535.31, 535.41, 535.51, 535.61, 535.71, 537.83, 537.84, 562.02, 562.03, 562.12, 562.13, 569.85, 578, 430, 431, 432.0, 432.1, 432.9, 852.0, 852.2, 852.4, 853.0, 336.1, 363.6, 372.72, 376.32, 377.42, 379.23, 593.81, 866.01, 866.02, 866.11, 866.12, 719.1, 729.92, 423.0, 772.5, | K22.6, K25-K28, K29.01, K29.21, K29.31, K29.41, K29.51, K29.61, K29.71, K29.81, K29.91, K31.811, K31.82, K52.81, K55.21, K56.60, K56.60, K57.01, K57.11, K57.13, K57.21, K57.31, K57.33, K57.81, K57.91, K57.93, K62.5, K92.0, K92.1, K92.2, I60, I61, I62, S06.340A-S06.349A, S06.350A-S06.359A, S06.360A-06.369A, S06.4X0A-S06.4X9A, S06.5X0A-S06.5X9A, S06.6X0A-S06.6X9A,  G95.11, G95.19, H05.23, H11.3, H31.3, H43.1, H47.02, I31.2, M25.0 N28.0, P54.4, S31.001A, S37.011A, S37.012A, S37.019A, S37.021A, S37.022A, S37.029A, S37.031A, S37.032A, S37.039A, S37.041A, S37.042A, S37.049A, S37.051A, S37.052A, S37.059A |
| **Gastrointestinal bleeding** | 530.7, 531, 531.2, 531.4, 531.6, 532, 532.2, 532.4, 532.6, 533, 533.2, 533.4, 533.6, 534, 534.2, 534.4, 534.6, 569.3, 535.01, 535.11, 535.21, 535.31, 535.41, 535.51, 535.61, 535.71, 537.83, 537.84, 562.02, 562.03, 562.12, 562.13, 569.85, 578 | K22.6, K25-K28, K29.01, K29.21, K29.31, K29.41, K29.51, K29.61, K29.71, K29.81, K29.91, K31.811, K31.82, K52.81, K55.21, K56.60, K56.60, K57.01, K57.11, K57.13, K57.21, K57.31, K57.33, K57.81, K57.91, K57.93, K62.5, K92.0, K92.1, K92.2 |

**ICD-9-CM** = International Classification of Diseases, Ninth Revision, Clinical Modification; **ICD-10-CM** = International Classification of Diseases, Tenth Revision, Clinical Modification.

**Table S3.** Diagnosis of top 10 indications for prescribing OCS burst.

| **Diagnosis** | ***n* (%)** |
| --- | --- |
| Dermatitis | 3,805 (7.55) |
| Acute upper respiratory infections | 1,925 (3.82) |
| Chronic obstructive pulmonary disease | 1,879 (3.73) |
| Acute bronchitis | 1,852 (3.68) |
| Urticaria | 1,367 (2.71) |
| Asthma | 1,027 (2.04) |
| Gout | 452 (0.90) |
| Acute nasopharyngitis [common cold] | 440 (0.87) |
| Acute sinusitis | 418 (0.83) |
| Pruritus | 417 (0.83) |

**OCS**: oral corticosteroid.

**Table S4.** Association of oral anticoagulants with major bleeding in patients with atrial fibrillation with cotherapy of OCS daily dose <10 mg versus OCS daily does >10 mg.

|  | **Daily dose <10 mg** | | |
| --- | --- | --- | --- |
|  | **CHR (95% CI)** |  | **AHR (95% CI)** |
| Warfarin + OCS burst | Ref |  | Ref |
| NOACs + OCS burst | 0.57 (0.52- 0.64) |  | 0.57 (0.52- 0.64) |
|  | **Daily dose >10 mg** | | |
|  | **CHR (95% CI)** |  | **AHR (95% CI)** |
| Warfarin + OCS burst | Ref |  | Ref |
| NOACs + OCS burst | 0.55 (0.48- 0.62) |  | 0.55 (0.49- 0.63) |

**NOACs**: non–vitamin K anticoagulants; **OCS**: oral corticosteroid; **CHR**: crude hazard ratio; **AHR**: adjusted hazard ratio; **CI**; confidence interval. ^*^Model was adjusted for age, sex, residence, income level, Charlson comorbidity index score, myocardial infarction, congestive heart failure, peripheral vascular disease, stroke, transient ischemic attack, dementia, chronic pulmonary disease, anemia, kidney diseases, and hepatic diseases, hypertension, bleeding history, and alcohol use, number of outpatient visits*,* non*-*steroidal anti*-*inflammatory drugs*,* proton pump inhibitors, antihypertensives, clopidogrel, ticlopidine, insulin, oral hypoglycemic agents, and lipid lowering agents.


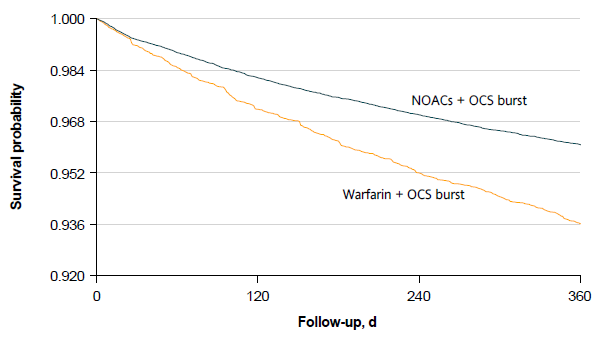


**Figure S1.** Kaplan-Meier curve for checking Cox proportional hazards assumption during 365-day follow-up in patients with atrial fibrillation treated various anticoagulants and OCS burst cotherapy. **OCS**: oral corticosteroid; **NOACs**: non–vitamin K anticoagulants.

**
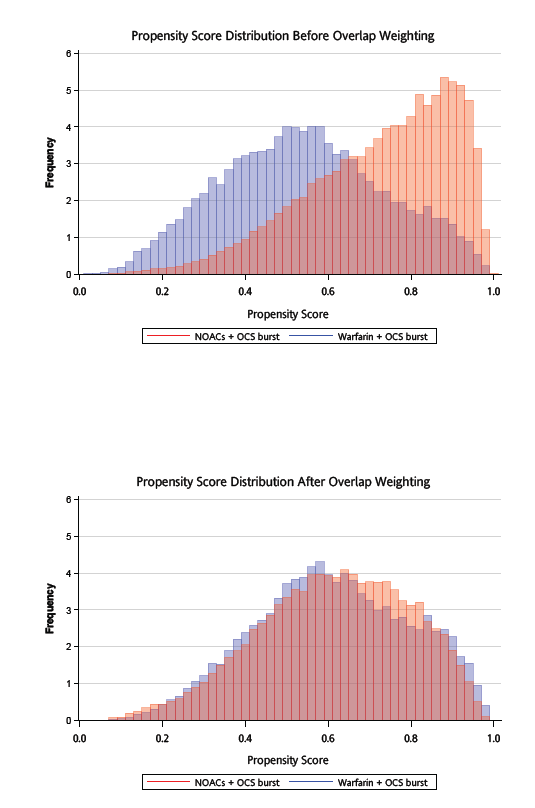
**

**Figure S2.** The distributions of propensity scores computed using baseline covariates before and after overlap weighting between patients with OCS burst cotherapy with NOACs versus warfarin.

**
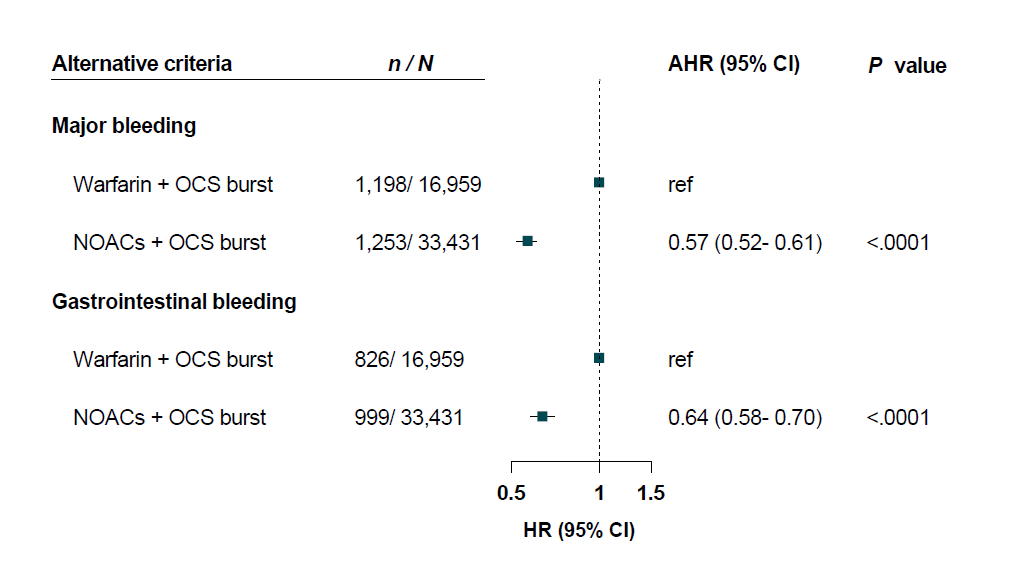
**

**Figure S3.** Association of oral anticoagulant and OCS burst cotherapy with major bleeding and gastrointestinal bleeding in patients with atrial fibrillation. **NOACs**: non–vitamin K anticoagulants; **OCS**: oral corticosteroid; **AHR**: adjusted hazard ratio; **CI**; confidence interval. ^*^Model was adjusted for age, sex, residence, income level, Charlson comorbidity index score, myocardial infarction, congestive heart failure, peripheral vascular disease, stroke, transient ischemic attack, dementia, chronic pulmonary disease, anemia, kidney diseases, and hepatic diseases, hypertension, bleeding history, and alcohol use, number of outpatient visits*,* non*-*steroidal anti*-*inflammatory drugs*,* proton pump inhibitors, antihypertensives, clopidogrel, ticlopidine, insulin, oral hypoglycemic agents, and lipid lowering agents.

**
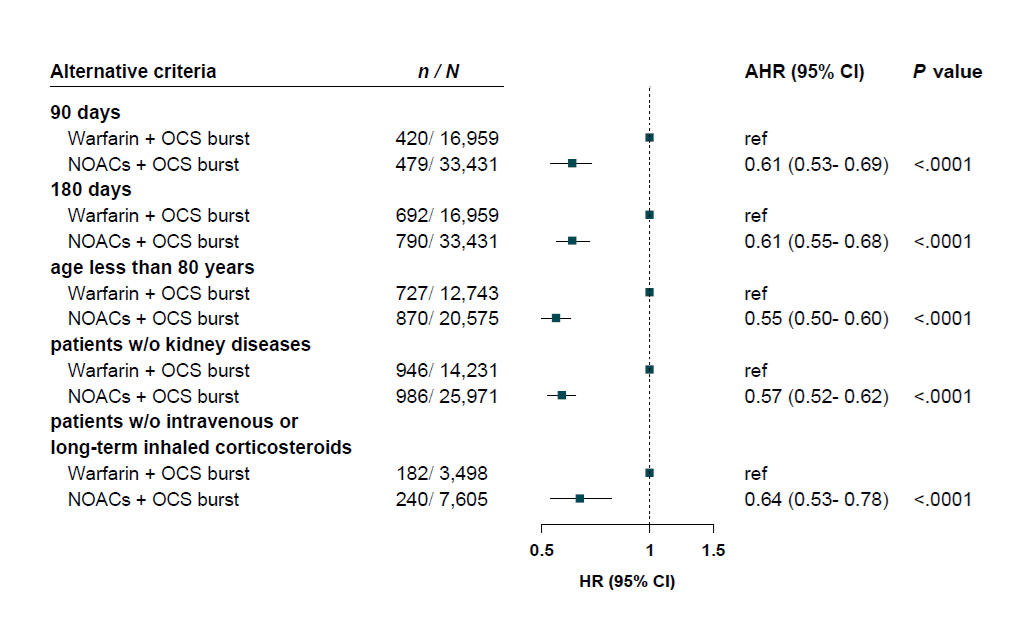
**

**Figure S4.** Association of oral anticoagulant and OCS burst cotherapy with major bleeding in patients with atrial fibrillation based on different follow-up periods: 90 and 180 days; patients aged less than 80 years; patients without kidney diseases; and patients without intravenous or long-term inhaled corticosteroid treatments. **NOACs**: non–vitamin K anticoagulants; **OCS**: oral corticosteroid; **AHR**: adjusted hazard ratio; **CI**; confidence interval. ^*^Model was adjusted for age, sex, residence, income level, Charlson comorbidity index score, myocardial infarction, congestive heart failure, peripheral vascular disease, stroke, transient ischemic attack, dementia, chronic pulmonary disease, anemia, kidney diseases, and hepatic diseases, hypertension, bleeding history, and alcohol use, number of outpatient visits*,* non*-*steroidal anti*-*inflammatory drugs*,* proton pump inhibitors, antihypertensives, clopidogrel, ticlopidine, insulin, oral hypoglycemic agents, and lipid lowering agents.
